# Hypoxia-Preconditioned Human Umbilical Cord Mesenchymal Stem Cells Transplantation Ameliorates Inflammation and Bone Regeneration in Peri-Implantitis Rat Model

**DOI:** 10.1055/s-0044-1791530

**Published:** 2024-11-07

**Authors:** Mefina Kuntjoro, Nike Hendrijantini, Eric Priyo Prasetyo, Bambang Agustono, Guang Hong

**Affiliations:** 1 Department of Prosthodontic, Faculty of Dental Medicine, Universitas Airlangga, Surabaya, Indonesia; 2 Department of Conservative Dentistry, Faculty of Dental Medicine, Universitas Airlangga, Surabaya, Indonesia; 3 Liaison Center for Innovative Dentistry, Graduate Scholl of Dentistry, Tohoku University, Aoba-Ku, Sendai, Japan

**Keywords:** dental implant, mesenchymal stem cell, peri-implantitis, bone regeneration, rat model, medicine

## Abstract

**Objective** The failure of dental implant treatments is predominantly attributed to peri-implantitis, which entails chronic inflammation within the peri-implant tissue, ultimately leading to tissue degradation. Addressing this condition, human umbilical cord mesenchymal stem cell (hUCMSC) transplantation serves as a regenerative therapy; however, concerns regarding the viability and efficacy of transplanted cells in inflamed regions persist. Hypoxic preconditioning of hUCMSCs has emerged as a potential strategy for augmenting their regenerative and immunomodulatory capacities. This study aimed to evaluate the expression of inflammatory (tumor necrosis factor [TNF]-α) and bone regenerative biomarkers (nuclear factor of activated T-cell [NFATc1], osteocalcin, collagen type I alpha 1 [COL1α1]) within peri-implantitis models subsequent to the transplantation of hypoxia-preconditioned hUCMSCs.

**Materials and Methods** Peri-implantitis models were established through the insertion of implants into the femur bone of 42 Wistar strain Rattus norvegicus, followed by intrasocket injection of lipopolysaccharide. The experimental animals were categorized into three groups (control, normoxia, and hypoxia) and underwent observation on days 14 and 28. The expression levels of TNF-α, NFATc1, COL1α1, and osteocalcin were evaluated using immunohistochemical staining, and the resulting data were subjected to one-way analysis of variance analysis ( *p* < 0.05).

**Results** Transplantation of hypoxia-preconditioned hUCMSCs significantly ameliorated inflammation and osteoclastogenesis, as evidenced by significant reductions in TNF-α and NFATc1 expression compared with the control group. Furthermore, hypoxic preconditioning of hUCMSCs demonstrated a significant elevation in the expression of osteocalcin and COL1α1 relative to the control group.

**Conclusion** The transplantation of hypoxia-preconditioned hUCMSCs exhibited a capacity to ameliorate inflammation and enhance bone regenerative processes in peri-implantitis rat models.

## Introduction

Peri-implantitis, a condition caused by periodontopathogenic bacterial plaque adhering to the peri-implant area and resulting in chronic inflammation, is a significant factor in dental implant failure. 1 The prominent bacteria involved is *Porphyromonas gingivalis* with their lipopolysaccharide (LPS) endotoxins which induce inflammation and bone destruction. 2 3 4 In this pathology, inflammation specifically affects the integrity of the peri-implant tissue, particularly the alveolar bone supporting the implant. One characteristic of peri-implantitis is increased osteoclastic activity, which leads to alveolar bone resorption and potential implant failure. 5 To address this issue, regenerative therapy involving mesenchymal stem cell (MSC) transplantation has rapidly developed in recent decades. Human umbilical cord is one of the most preferred sources in harvesting MSCs due to their noninvasive nature. 6 7 Human umbilical cord mesenchymal stem cells (hUCMSCs) maintain their stem cell characteristics even after undergoing multiple passages and expansion. The surface antigens of hUCMSCs are not highly noticeable, resulting in minimal rejection of transplanted cells. Moreover, the requirements for matching hUCMSCs in allografts and scaffolds are not excessively strict, making them easier to use in such procedures. 8 9

hUCMSCs have shown great potential in repairing tissues and renewing damaged cells through differentiation, immune regulation, and paracrine processes. A previous study conducted by Hendrijantini et al reported that this cell type exhibits a high osteogenic property, as evidenced by its ability to induce endogenous osteoblastogenesis activity in an animal model of osteoporosis. 10 This cells also have promising use in systemic conditions such as diabetes mellitus. 11 12 Despite the demonstrated efficacy of hUCMSCs in stimulating bone regeneration, their therapeutic application is constrained by their limited viability within inflamed host tissues. A previous study reported that preconditioning hUCMSCs, adipose MSCs (AMSCs), and gingival MSCs (GMSCs) with hypoxic condition can significantly enhance their adaptive potential by upregulating the expression of hypoxia-inducible factor (HIF)-1α, thereby correlating with enhanced stemness characteristics and overall adaptability of the hUCMSCs, AMSCs, and GMSCs. 13 14 15 Moreover, investigations have provided evidence that preconditioning MSCs under hypoxic conditions improve their regenerative and immune regulatory properties. 16

Building upon this information, the objective of this study was to explore the effectiveness of hypoxia-preconditioned hUCMSCs in mitigating the pathological processes associated with peri-implantitis. This investigation focused on evaluating various inflammatory and osteogenic biomarkers.

## Materials and Method

### Study Design and Setting

This study was conducted in the Laboratory of Animal Trial, Institute of Tropical Disease, Universitas Airlangga, Surabaya, Indonesia. Faculty of Dental Medicine, Universitas Airlangga, Surabaya, Indonesia assigned this study protocol ethical approval for animal laboratories with number 959/HRECC.FODM/XII/2022. This research was conducted following ethical standards of experiments. This research was a true experimental laboratory study using an analytical posttest control group design. Lemeshow's formula determines the minimum sample; the total replication needed is 42, with 7 replications for each group. The groups consist of: K1 (control 14 days), K2 (control 28 days), PN1 (normoxia hUCMSCs 14 days), PN2 (normoxia hUCMSCs 28 days), PH1 (hypoxia hUCMSCs 14 days), and PH2 (hypoxia hUCMSCs 28 days). Male Wistar rats ( *Rattus norvegicus* ) weighing 325 to 350 g and 8 to 10 weeks old made up the sample. All of the experimental animals were free of any oral mucosal and systemic pathologies.

### Implant Type and Size

Cylindrical grade 1 titanium implants with 2 mm height and 1 mm diameter, fabricated by computer-aided design/computer-aided manufacturing machine (Yoshimi Inc. Osaka, Japan), were used in the study.

### Peri-Implantitis Rat Model

The rats underwent a preoperative fasting period of 6 to 8 hours. Anesthesia was induced through intramuscular injection of a combination of ketamine (10% solution, 1 mL) and xylazine (1 mL) into the semitendinosus muscle located in the gluteus region. Prior to the surgical procedure, depilation of the femoral area where the implant was to be inserted was performed, followed by disinfection using iodine soap and 80% alcohol. Sterilization of surgical instruments was performed using an autoclave.

The surgical procedure was performed according to previously published method. 11 In brief, a 10-mm incision on the dorsal surface of the femur until the bone surface was exposed. Sequential drilling was conducted, considering the appropriate length and diameter of the implant, and utilizing saline irrigation at a maximum rotational speed of 1,500 revolutions per minute (rpm). The implant was carefully positioned in the osteotomy site, approximately 1 cm from the mesial head of the femur, ensuring the attainment of primary stability. Evaluation of primary stability was performed using a tapping device matching the implant diameter to confirm its immobility.

To induce peri-implantitis, the commercially available Invivo Gen LPS-PG Ultrapure 1 mg was employed. The LPS stock solution was prepared by diluting 1 mg of LPS in 10 mL of phosphate-buffered saline (PBS), resulting in a concentration of 1 µg/0.01 mL of PBS. Subsequently, 0.01 mL of LPS solution was administered into the implant socket area via a Hamilton syringe prior to implant placement. Following LPS induction, the implant was inserted above the LPS solution, and the surrounding muscles and skin were sutured using 4–0 vicryl material, and waited for 4 weeks to induce a peri-implantitis model.

### Human Umbilical Cord Mesenchymal Stem Cells Preparation

The hUCMSCs were sourced from the umbilical cord of infant born at Dr. Soetomo General Hospital in Surabaya, East Java, Indonesia with appropriate parental consent, in accordance with the Declaration of Helsinki. The informed consent signed by the donor and the umbilical cord collection was approved by the Dr. Soetomo Hospital Medical Committee (Permit number: 547/Panke. KKE/IX/2017).

Following an established protocol, the MSCs were isolated and cultured until they reached a confluence of 70 to 80%, corresponding to the 6th passage. Hypoxic conditions were induced using an indirect approach involving the application of a solution containing cobalt (II) chloride hexahydrate (CoCl2 • 6H2O) with a molecular weight of 237.9. Prior to use, the CoCl2 solution was diluted with sterile water to achieve a concentration of 100 μM. The cell culture was then supplemented with the prepared CoCl2 solution and incubated at 37°C in a CO2 incubator for 48 hours, maintaining a 5% CO2 environment.

Four weeks after peri-implantitis model induction, incision at the implant site was done to access the peri-implantitis. Stabident (Henry Schein, United States) was drilled at 800 rpm and 20N torque to make perforation into the bone. The 30-µL hUCMSCs (500,000 cells) were injected using 27G syringe into the bone 1 mm from the proximal and distal side of the implant for each animal. For the control group (K1 and K2), gelatin only was injected into the perforated bone. The incision was sutured by absorbable suturing material. 11

### Biomarkers Analysis

Following the completion of the treatment phase, the experimental animals were euthanized, and the femur bones were collected for histopathological preparations. The histopathology slides underwent immunohistochemical processing using horseradish-labeled monoclonal antibodies targeting tumor necrosis factor (TNF)-a, nuclear factor of activated T-cell (NFATc1), osteocalcin, and collagen type I alpha 1 (COL1α1). In addition, 3–3′ diaminobenzidine (Abcam, United States) was utilized as a substrate to visualize the positive protein expression, resulting in a brown precipitate within the cells of the implant site. This precipitate was subsequently observed and analyzed using an inverted light microscope, with magnifications of 400× across five different fields of view. The observations were made by two independent pathologists.

### Statistical Analysis

IBM SPSS Statistics 26 (for Windows, v9.4.1, Chicago, Illinois, United States) was used to perform the statistical analysis. The Shapiro–Wilk test was employed to check the normality of the data, while Levene's test was used to assess the homogeneity. If the data satisfied the assumptions for parametric tests, a one-way analysis of variance (ANOVA) test with a significance level of *p* < 0.05 was conducted to identify differences among all groups. Conversely, if the data did not meet the assumptions for a parametric test, the nonparametric Kruskal–Wallis test ( *p* < 0.05) was employed. Subsequently, Tukey's honestly significant difference post hoc test was performed to identify specific differences between groups.

## Results

This study examined the expression of NFATc1, osteocalcin, TNF-α, and COL1α1. It was found that NFATc1 and osteocalcin data demonstrated homogeneity and conformed to a Gaussian distribution. However, TNF-α and COL1α1 data did not meet these assumptions. Therefore, a one-way ANOVA was performed to analyze the NFATc1 and osteocalcin data, while the Kruskal–Wallis test was employed for the TNF-α and COL1α1 data. The significance level was set at *p* < 0.05 for all tests.

### NFATc1 Expression

**Fig. 1:**
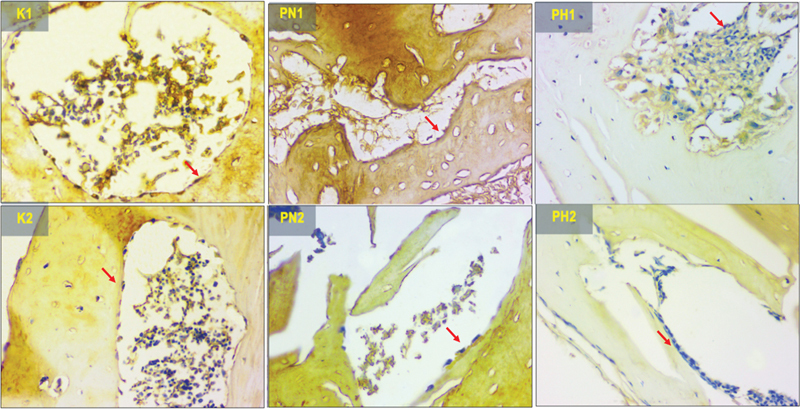
The histological view of nuclear factor of activated T-cell (NFATc1) expression in 400× magnification (the protein expression represented by the red arrows). K1 (control 14 days), K2 (control 28 days), PN1 (normoxia human umbilical cord mesenchymal stem cells [hUCMSCs] 14 days), PN2 (normoxia hUCMSCs 28 days), PH1 (hypoxia hUCMSCs 14 days), and PH2 (hypoxia hUCMSCs 28 days).

Fig. 1 presents the histopathology view illustrating the NFATc1 expression pattern at the implant site. The bony wall of the implant site exhibits brown precipitates, indicating NFATc1 expression. The one-way ANOVA results, as shown in Table 1 , reveal statistically significant differences among all groups ( *p* = 0.000). Significant difference ( *p* < 0.05) was observed between the control group (K1) and PN2, PH1, and PH2. Significant difference was also found in control group (K2) and PH2. The lowest NFATc1 expression was found in the hypoxia group (PH2).

**Table 1:** The expression of NFATc1, TNF-α, osteocalcin, and COL1α1 in peri-implantitis rat model

Observation days	Group	TNF-α	NFATc1	Osteocalcin	COL1α1
		Mean ± SD			
14 days	Control	9.77 ± 1.54 ^a,b,c^	7.97 ± 1.96 ^a,b,c^	3.04 ± 1.47 ^a,b,c,j^	2.86 ± 0.70 ^a,b,c,d,e^
	Normoxic hUCMSCs	8.57 ± 1.38 ^g,h^	5.00 ± 2.62	5.40 ± 0.98 ^b,g,h,i^	5.86 ± 0.80 ^b^
	Hypoxic hUCMSCs	5.43 ± 2.77 ^b,e^	2.94 ± 1.44 ^b^	8.80 ± 2.11 ^e,h,j^	6.66 ± 0.64 ^d,f^
28 days	Control	8.60 ± 1.43 ^d,e,f^	5.66 ± 2.01 ^d^	3.64 ± 1.77 ^d,e,f^	4.53 ± 0.75 ^a,e,f,g^
	Normoxic hUCMSCs	5.48 ± 1.90 ^a,d,g^	4.40 ± 2.31 ^a^	8.96 ± 1.32 ^a,d,g^	6.46 ± 0.30 ^c,e^
	Hypoxic hUCMSCs	4.48 ± 1.40 ^c,f,h^	2.28 ± 1.00 ^c,d^	10.72 ± 1.23 ^c,f,h^	8.20 ± 1.44 ^e,g^
*p* -Value		0.000 k	0.000 k	0.000 k	0.000 k

Abbreviations: ANOVA, analysis of variance; COL1α1, collagen type I alpha 1; hUCMSCs, human umbilical cord mesenchymal stem cells; NFATc1, nuclear factor of activated T-cell; SD, standard deviation; TNF-α, tumor necrosis factor-α.

Note: The same letter represents significant difference between the values within the variable.

^a–j^ Represent significance difference in mean and standard deviation between compared groups.

k Significant difference of ANOVA and Kruskal–Wallis represented by *p* < 0.05.

### TNF-α Expression

**Fig. 2:**
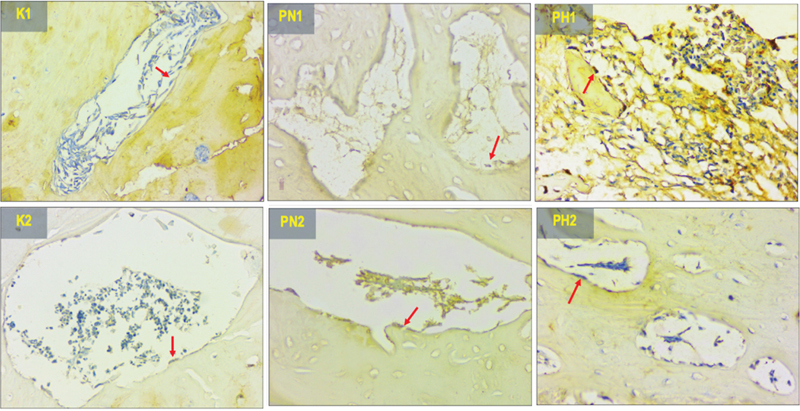
The histological view of tumor necrosis factor (TNF)-α expression in 400× magnification (the protein expression represented by the red arrows). K1 (control 14 days), K2 (control 28 days), PN1 (normoxia human umbilical cord mesenchymal stem cells [hUCMSCs] 14 days), PN2 (normoxia hUCMSCs 28 days), PH1 (hypoxia hUCMSCs 14 days), and PH2 (hypoxia hUCMSCs 28 days).

Fig. 2 depicts the presence of brown precipitates in the bone, indicating the TNF-α expression pattern at the implant site. The Kruskal–Wallis test revealed significant differences among all groups ( *p* = 0.0001), as presented in Table 1 . Significant difference ( *p* < 0.05) was observed between the treatment groups (PN1, PN2, PH1, PH2) and the control group (K1, K2). In the normoxic groups (PN1, PN2) TNF-α was significantly lower, while in the hypoxic groups (PH1, PH2) no significant different was found. Meanwhile, between PN2, PH1, and PH2 there were no significant differences. This proved that lower TNF-α can be reached in 14 days in hypoxia groups.

### Osteocalcin Expression

**Fig. 3:**
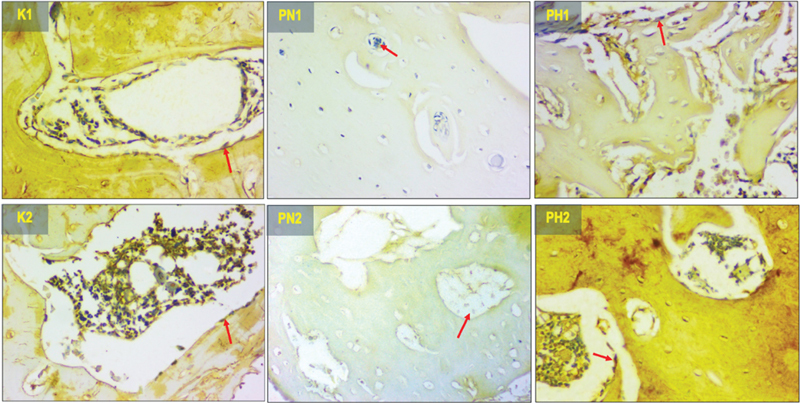
The histological view of osteocalcin expression in 400× magnification (the protein expression represented by the red arrows). K1 (control 14 days), K2 (control 28 days), PN1 (normoxia human umbilical cord mesenchymal stem cells [hUCMSCs] 14 days), PN2 (normoxia hUCMSCs 28 days), PH1 (hypoxia hUCMSCs 14 days), and PH2 (hypoxia hUCMSCs 28 days).

The expression pattern of osteocalcin at the implant site is depicted in Fig. 3 through histopathological examination. The results of the one-way ANOVA, presented in Table 1 , indicate significant differences among all groups ( *p* = 0.000). Significant difference ( *p* < 0.05) was observed between the treatment groups (PN1, PN2, PH1, PH2) and the control groups (K1, K2). In the normoxic groups (PN1, PN2) osteocalcin was significantly higher, while in the hypoxic groups (PH1, PH2) no significant difference was found. Meanwhile, between PN2, PH1, and PH2 no significant difference was revealed. This proved that higher osteocalcin can be reached in 14 days in hypoxia groups, while in normoxia it required 28 days.

### COL1α1 Expression

**Fig. 4:**
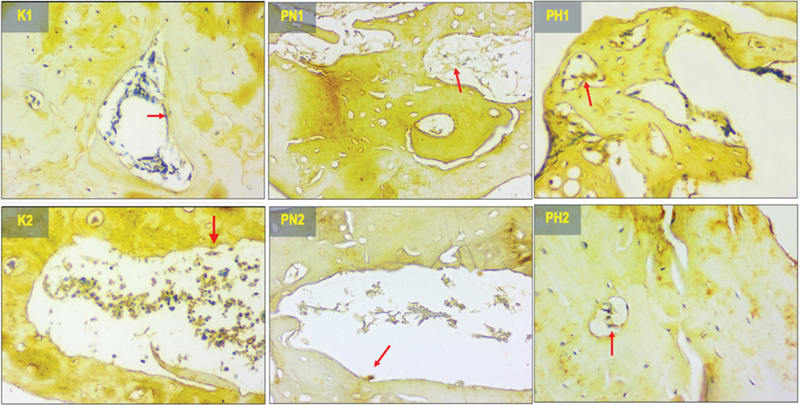
The histological view of collagen type I alpha 1 (COL1α1) expression in 400× magnification (the protein expression represented by the red arrows). K1 (control 14 days), K2 (control 28 days), PN1 (normoxia human umbilical cord mesenchymal stem cells [hUCMSCs] 14 days), PN2 (normoxia hUCMSCs 28 days), PH1 (hypoxia hUCMSCs 14 days), and PH2 (hypoxia hUCMSCs 28 days).

The expression pattern of COL1α1 at the implant site is illustrated in Fig. 4 , where brown precipitates signify its presence in the bone. The Kruskal–Wallis test yielded statistically significant differences among all groups ( *p* = 0.0001), as outlined in Table 1 . Significant difference ( *p* < 0.05) was observed between the treatment groups (PN1, PN2, PH1, PH2) and the control groups (K1, K2). In the normoxic groups (PN1, PN2) COL1α1 was significantly higher, while in the hypoxic groups (PH1, PH2) no significant difference was found. Interestingly, K1 was significantly lower compared with all groups, while K2 was significantly lower compared with PN2, PH1, and PH2. This proved that hypoxia leads to higher COL1α1 expression 14 days sooner than in normoxic which needs 28 days.

## Discussion

Peri-implantitis is characterized by dysregulated immune response and disruption of the homeostatic state of the bone microenvironment. 17 The presence of LPS, a prominent contributor to peri-implantitis, elicits immune response and promotes osteoclastogenesis. 18 LPS instigates immune response activation by engaging various pattern recognition receptors expressed on innate immune cells, including Toll-like receptor-4 and -2. 19 This interaction initiates inflammatory signaling cascades, such as nuclear factor kappa B (NF-κB) and mitogen-activated protein kinase, culminating in the secretion of diverse inflammatory cytokines (TNF-α, interleukin [IL]-1β, IL-6), and the modulation of NFATc1, c-Fos, c-Jun N-terminal kinases, and extracellular signal-regulated kinases. Additionally, LPS influences osteoclast maturation by modulating the activity of matrix metalloproteinase-9. 20

MSCs possess considerable potential in tissue repair, promote cell survival and growth factors, and chemoattractant ability to the injury site. 21 Furthermore, emerging hypotheses propose that the regenerative characteristics of MSCs stem from their ability to generate diverse bioactive molecules that exert cytoprotective, immunotropic, and proregenerative effects. 22 23 Nevertheless, the clinical application of MSC therapy encounters limitations linked to the survival challenges faced by MSCs within unfavorable host tissue environments, particularly in the presence of inflammation. Inflammatory conditions give rise to the accumulation of various molecules, including reactive oxygen species and cytotoxic factors, which pose threats to the viability of MSCs. 22 24 These limitations underscore the necessity for techniques that can manipulate the properties and capabilities of MSCs during the pretransplantation stage.

Preconditioning MSCs with hypoxia during the culture phase has demonstrated the ability to enhance their viability following transplantation in unfavorable tissue conditions. 25 26 Culturing MSCs under 5% hypoxic conditions stimulates their proliferation potential, preserves their properties, inhibits senescence, and enhances their osteogenic differentiation capacity. 27 Similarly reported that hypoxic preconditioning of GMSCs using cobalt chloride (CoCl2) improves the adaptive capacity of GMSCs after transplantation into tissues. The study revealed that hypoxic preconditioning significantly upregulates the expression of HIF-1α, a transcription factor associated with heightened stemness properties and adaptability of stem cells. 15 *In vitro* study using hUCMSCs pretreated hypoxia revealed significantly higher HIF-1 α and mammalian target of rapamycin (mTOR) expression. HIF-1α is a main marker that promotes proliferation and cell metabolism, while mTOR connects metabolism affected by hypoxia and increases hUCMSCs migration, proliferation, and survival. 25 This finding aligns with previous studies that have demonstrated enhanced posttransplantation viability of hypoxia-preconditioned stem cells compared with normoxia. 28 Furthermore, hypoxic preconditioning has been shown to enhance the secretion of angiogenic factors, anti-inflammatory cytokines, and micro-ribonucleic acids by MSCs, further reinforcing their capacity for tissue repair. 16 29 30

Many sources for MSCs have been investigated; umbilical cord shows superior capacity than others through their proliferation, differentiation, and immunomodulation ability. 11 Recently, hUCMSCs researches have been conducted to prove their differentiation into osteoblastic lineage. Previous hUCMSCs *in vivo* study shows significantly higher osteoblastogenesis activity such as transforming growth factor-β1, Runx2, and the number of osteoblast cells. 10 The induction of hUCMSCs also decreased osteoclastic activity, therefore bone formation and regeneration could be increased. 11

The present study provides evidence that hypoxic preconditioning of hUCMSCs can effectively reduce inflammatory and osteoclastic activity, as demonstrated by the significant decrease in TNF-α and NFATc1 expression compared with the control group. TNF-α is a key proinflammatory cytokine implicated in peri-implantitis, known for its role in activating innate immune cells such as macrophages and neutrophils. Moreover, TNF-α have the potential to indirectly stimulate bone resorption by influencing the synthesis of a crucial factor called receptor activator of NF-κB ligand (RANKL) and its counterpart, osteoprotegerin (OPG), which acts as a decoy receptor. This influence can occur through their impact on osteoblast/stromal cells. Additionally, these cytokines can directly promote the growth and/or function of cells in the osteoclast lineage, which contributes to the process of resorption. 31 32 Lower TNF-α in this study can be reached faster (14 days) in hypoxia-pretreated hUCMSCs than normoxia groups (28 days).

This study confirmed the antiosteoclastic activity of hypoxia-preconditioned hUCMSCs shown by a substantial reduction in NFATc1 expression compared with the control group. NFATc1 serves as a crucial regulator of osteoclast differentiation and is involved in regulating the activity of osteoclastic genes such as thrombin receptor activator protein and cathepsin K. 33 These findings demonstrate the capacity of hUCMSCs in preventing bone resorption in peri-implantitis. Hypoxic and normoxic hUCMSCs both effectively decrease NFATc1. In hypoxia groups, lower NFATc1 can be reached in day 14, while in the normoxia group it required 28 days. This proves that the hypoxia precondition leads to decreased osteoclastogenesis activity.

This study investigated the efficacy of hypoxia-preconditioned hUCMSCs in facilitating alveolar bone regeneration. The participation of COL1α1 in promoting the attachment and proliferation of osteoblasts on the implant surface expedites the development of new bone surrounding the implant. 34 Consequently, this mechanism exerts a significant influence on the treatment's effectiveness and the long-term stability of the implant. This study revealed significant differences between treatment groups, both the normoxia or hypoxia hUCMSCs compared with the control group across both observation days, indicating that hUCMSCs administration accelerates COL1α1 deposition in the peri-implant region. Surprisingly, higher COL1α1 expression was reached in 14 days earlier in hypoxia groups compared with the normoxic group. No difference between the hypoxia groups happened because the COL1α1 expression still needed to maintain the collagen deposition and mineralization phase. 35 This study is relevant with previous study that stated that COL1α1 after hUCMSCs application remain stable after 4 and 8 weeks. 36

This study examined the expression of osteocalcin, a prominent noncollagenous protein abundant in bone, which is synthesized by mature osteoblasts during late-stage osteogenesis. Osteocalcin, a noncollagenous protein in bone extracellular matrix, has been identified to play a significant role in osteogenic differentiation of MSCs. 37 Thus, osteocalcin serves as a vital biological marker for assessing the late stage of osteogenesis. In line with this, the study observed increased osteocalcin expression in hypoxia-preconditioned hUCMSCs compared with the control group. Moreover, a noteworthy finding was the significantly higher expression of osteocalcin in the hypoxia-preconditioned hUCMSCs group compared with the normoxia-preconditioned group on the 14th day of observation. This outcome indicates that hypoxia preconditioning enhances the regenerative and osteogenic capacities of hUCMSCs, facilitating the accelerated attainment of the final stage of osteogenesis compared with normoxia preconditioning. These findings are consistent with a previous study which demonstrated MSCs from gingiva cultured under hypoxia exhibit an increased potential for osteogenic differentiation and resulted a significant elevation in the expression of osteopontin. 38 Observation on 28th day revealed no significance between the normoxia and hypoxia group. This condition is related to the peak of osteocalcin expression that happened on the second week. 39

## Conclusion

The transplantation of hypoxia-preconditioned hUCMSCs demonstrated the ability to alleviate inflammation and promote bone regeneration in a rat model of peri-implantitis. Hypoxia hUCMSCs leads to the acceleration of osteoblastogenesis and decreased inflammation compared with normoxia from 28 to 14 days. This study implicates the suitability of hypoxia-preconditioned hUCMSCs in various clinical application in dentistry, mainly in periodontics, implantology, prosthodontics, regenerative endodontics, and oral and maxillofacial surgery. However, further *in vivo* investigations involving the analysis of biomarkers related to osteogenesis to further confirm the results in rat and higher-level animal models are needed.
